# Effect of Mg on the Structural, Optical and Thermoluminescence Properties of Li_3_Al_3_(BO_3_)_4_: Shift in Main Glow Peak

**DOI:** 10.3390/molecules28020504

**Published:** 2023-01-04

**Authors:** Adil Alshoaibi, Patrick O. Ike, Assumpta C. Nwanya, Chawki Awada, Shumila Islam, Fabian I. Ezema

**Affiliations:** 1 Department of Physics, College of Science, King Faisal University, Al Ahsa 31982, Saudi Arabia; 2 Department of Physics and Astronomy, University of Nigeria, Nsukka 410001, Nigeria; 3 Faculty of Natural Sciences, Caritas University, Amorji-Nike, Enugu 400103, Nigeria; 4 Nanosciences African Network (NANOAFNET) iThemba LABS-National Research Foundation, Somerset West 7129, South Africa; 5 UNESCO-UNISA Africa Chair in Nanosciences/Nanotechnology, College of Graduate Studies, University of South Africa (UNISA), Pretoria 0002, South Africa; 6 Africa Centre of Excellence for Sustainable Power and Energy Development (ACE-SPED), University of Nigeria, Nsukka 410001, Nigeria

**Keywords:** dopant, dosimetry, lithium aluminium borate, magnesium, structure, thermoluminescence

## Abstract

The doping of magnesium on lithium aluminium borate phosphor is reported in this study. A solid-state sintering technique was employed as the borate samples were synthesized. This report focuses on the structural, optical, thermoluminescence, and kinetic analyses of the main glow peak. The structural properties of lithium aluminium borates improved due to the magnesium dopants used. Differences in the crystallite size and particle size were 38.85–67.35 nm and 50–60 nm, respectively, and these results were obtained from the analyzed X-ray diffractogram and scanning electron spectroscopy. The energy band gaps obtained from the direct transition of borate phosphor materials were within the range of 3.00–4.40 eV, and the doped samples gave a higher energy band gap. A decrease in the TGA (%) exhibited a weight loss or water loss for the undoped, 0.1% Mg, and 0.3% Mg-doped lithium aluminium borate materials. The glow curve measured at a heat rate of 1 °C·s^−1^ after irradiation to 50 Gy revealed four peaks related to the magnesium doped lithium aluminium borate. The main glow peak was observed at 86 °C. Activation energy was extracted from the main glow peak by using kinetic analysis which involves the initial rise, deconvolution, and variable heating rate approach, and it was approximately 0.67 ± 0.03 eV. A shift in the main glow peak curve from 86 to 110 °C was recognized for the magnesium-doped lithium aluminium borate when it was irradiated from 1 to 300 Gy.

## 1. Introduction

The structural, thermal, and chemical stability of lithium aluminum borate materials have encouraged their usein radiation protection, laser devices, scintillators, and thermoluminescence studies [1,2,3,4,5]. These properties are strongly associated with the structure of materials and are of the utmost interest to researchers because they are necessary for dosimetry.

The structural behavior of anionic groups of borate composites which consist of boron and oxygen atoms is important regarding the discussion of thermoluminescence dosimetry (TLDs). Some classifications of borate crystal structures described [6,7,8,9] and reviewed in various publications [10,11,12,13,14,15,16] are referred to as boron and oxygen atom coordinates which are interconnected in triangular (BO_3_) and tetrahedral (BO_4_) borates. Such an alliance is associated with joint oxygen atoms which constitute the fundamental building blocks (FBB) of most borate structures [17,18,19,20,21,22,23]. The highest number of borates based on triangular (BO_3_) and tetrahedral (BO_4_) coordinates are found in the distribution of the topological structure [12]. These structural formations of boron–oxygen found in lithium aluminium borate have led to stable compounds that do not vary considerably in different crystals and glass ceramics [24].

Structural studies on lithium aluminium borate have long been pursuedin order to develop materials with good thermal and chemical stability that can be applied in various fields. The phase structure of lithium aluminium borate (Li_4_Al_4_B_6_O_17_) ceramic was first reported by Kim and Hummel [25]. The crystal structure of the lithium aluminium borate (Li_2_O–Al_2_O_3_–B_2_O_3_) ternary system was later investigated by He et al. [26], and the microwave dielectric ceramic of lithium aluminium borate (Li_3_AlB_2_O_6_) was reported by Ohashi et al. [27]. Aluminium, which is similar to boron, is commonly in tetra-coordinationand often performs like a tetrahedral coordinate to boron in compounds [26]. If an aluminium atom can be replaced with a tetra-coordinated boron atom, new structures of lithium aluminium borate compounds can be produced [26]. The ternary system of other lithium aluminium borate structures identified as Li_2_Al_4_B_4_O_13_, Li_4_Al_2_B_4_O_11_, Li_2_AlBO_4_, Li_2_Al_2_B_2_O_7_, Li_2_Al_2_B_4_O_10_, and Li_3_AlB_2_O_6_ were studied in more detail by Abdullaev et al. [28]. From all the identified structures, only Li_3_AlB_2_O_6_ was structurally characterized and compared with all the aforementioned lithium aluminium borate compounds [29,30].

Alkaline earth elements employed as dopants are reported to be soluble in most borate materials [31]. These elements usually engage in compounds to improve the TL yield and optical properties, which include sensitivity, dose–response, absorbance, and low fading. In addition, the ability to adjust the properties of lithium aluminium borates allows them to successfully serve in various applications. Alkaline earth metals that are commonly used as dopants to achieve the aforementioned properties in luminescence studies are magnesium (Mg), calcium (Ca), barium (Ba), strontium (Sr), and beryllium (Be). The luminescence, morphology, optical properties, and efficiency of lithium aluminium borate compounds can be enhanced by modifying the concentration of magnesium. In this study, the alkaline earth metal magnesium was doped lithium aluminium borate in order to explore its TL and optical properties and thus its application regarding radiation dosimetry.

The aim of this article is to study the effect of magnesium on lithium aluminium borate which was synthesized by adopting the solid-state sintering method. Structural properties were analyzed using X-ray diffraction (XRD) and a scanning electron microscope (SEM). The absorbance and energy band gap, which share details about optical properties, were obtained using UV–Vis spectroscopy. A thermoluminescence reader was used to study the thermoluminescence properties. A kinetic analysis of the phosphor with the best magnesium sensitivity doped on the lithium aluminium borate was determined based on the TL glow curve.

## 2. Experimental Route

The solid-state sintering technique was adopted for the synthesis of lithium aluminium borate (Li_3_Al_3_(BO_3_)_4_) samples. Analytical grade precursors (98%) from Sigma Aldrich were used at a given mass of 1.64 g for lithium carbonate, 3.46 g for aluminium hydroxide, and 3.67 g for boric acid. The precursors were mixed in agitate mortar by adding 50 mL of deionized water, which was then stirredat 40 revolutions per minute for 30 min to obtain a homogeneous distribution of stochiometric compounds. Other baths were obtained by adding various masses of 0.34, 0.76, 1.10, 1.40, and 1.78 mg magnesium dopant, which corresponded to mole concentrations of 0.1%, 0.2%, 0.3%, 0.4%, and 0.5%, respectively. Then, the resultant solution was heated and dried on a hot plate at about 100 °C inside a fume chamber for about 30 min. The obtained mixed material was then heated in a muffle furnace (Searchtech Instruments SXL) at 950 °C for a period of 5 h. The samples were removed from the furnace when they were at a molten state from; next, theywere exposed and allowed to cool to an ambient temperature. The hard whitish ceramic materials were crushed into a fine powder before they were placed back into the muffle furnace and further annealed to a temperature of 300 °C for an hour. The high temperature annealing removed water molecules and carbon dioxide which helped form a stable material, and further annealing was done on the powdered samples before further studies were carried out to improve its structural and luminescence properties.

The morphological structure was determined using a scanning electron microscope (ZEISS GeminiSEM 500, NanoFab Microscopy, Gaithersburg, MD 20899-6201, USA). The grain size arrangement from the obtained morphological structure was also analyzed with imagej freeware. An X-ray diffractometer (AXS D8 diffractometer, Bruker, Allentown, PA, USA) was used to study the crystal structure. A visible spectrum was used to determine the UV–vis–NIR spectra (UV-1800 Shimadzu UV spectrophotometer, Mettler-Toledo, Columbus, OH 43240, USA) from which the optical properties were obtained. Thermal analysis (TG-DTA and TMA Rigaku Evo plus II, Tokyo, Japan) was used to study the weight loss of the compounds. Thermoluminescence of all the samples was measured using an A RISØ TL/OSL DA-20 Luminescence Reader (DTU Risø Campus, DK-4000 Roskilde, Denmark). The samples were properly irradiated using a ^90^Sr/^90^Y β source at a time rate of 0.1 Gy/s. Luminescence was recognized using an EM 9235QB Photomultiplier tube that was fitted through a 7.0 mm Hoya U-340 filter. All the measurements that were detected were created at a heating rate of 1 °C/s. Kinetic analyses were also studied based on the obtained TL results.

## 3. Results and Discussions

### 3.1. Structural Analysis

**Figure 1:**
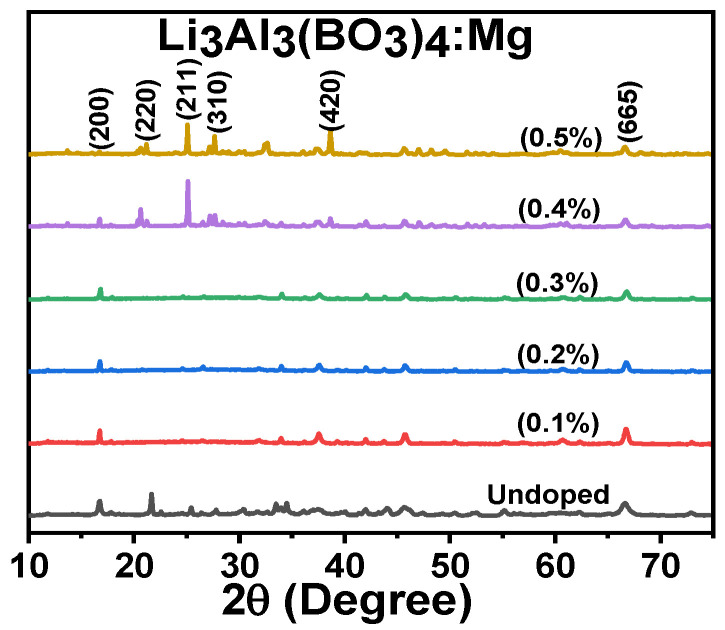
The XRD outline for the Mg-doped and undoped lithium aluminium borate.

XRD plots at a 2θ angle from 10 to 75° that were designed for both the undoped concentrations and the various concentrations of magnesium doped lithium aluminium borate phosphor and are shown in Figure 1. Peak intensities were observed from all the samples, but they appear to be more intense at higher concentrations of the magnesium doped (0.4% and 0.5%) samples. The 2θ value from the indicated intensity peaks at 17.06°, 22.08°, 24.67°, 26.80°, 38.23°, and 66.90°correspond to the planes (200), (220), (211), (310), (420), and (665), respectively. The structure from the XRD outline of Li_3_Al_3_(BO_3_)_4_ coordinated with the JCPDS card number 00-015-0344.

The corresponding crystallite sizes (*S*) for all the samples were calculated based on the most consequential intense peak at a 2*θ* angle of 17.06 ± 0.03° by using Debye Scherrer’s equation [32]:

(1) $$S=\frac{0.9\lambda }{\beta \mathrm{Cos}\theta }$$

where *λ* = 1.5406Å is the wavelength for the X-ray target; *θ* is Bragg’s angle; and *β* is the full width at half maximum intensity. The calculated crystallite size of the undoped lithium aluminium borate was 53.95 nm, as shown in Table 1. There was a gradual increase in the crystallite size from 0.1% to 0.5% mole concentration for the Mg-doped lithium aluminium borate, as shown in Table 1. The crystallite size for concentrations of 0.1%, 0.2%, and 0.3% Mg-doped lithium aluminium borate were less than the value of 53.95 nm for the undoped lithium aluminium borate, while the 0.4% and 0.5% mole concentrations were greater than 53.95 nm. The regular crystal size increased as the mole concentration of the magnesium dopant increased. A saturation phase was reached just before 0.4% to 0.5% mole concentrations, and a further increase resulted in an increase in the crystallite size which was observed to be greater than the undoped material of lithium aluminium borate. This could be as a result of the reaction as it was no longer effective during the mix up.

### 3.2. Morphology Survey

**Figure 2:**
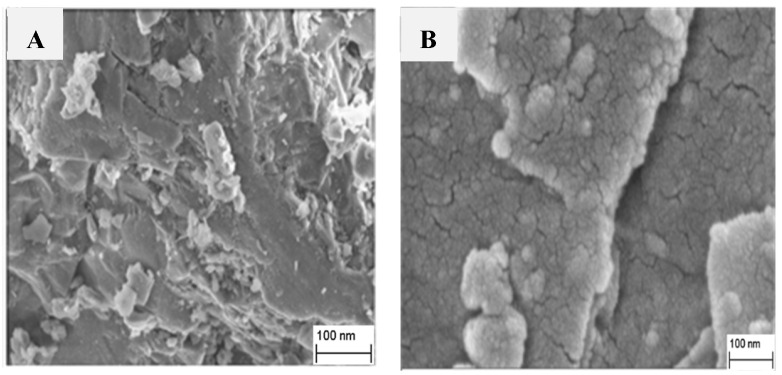
SEM micrographs of (**A**) undoped lithium aluminium borate and (**B**) 0.1% magnesium-doped lithium aluminium borate.

Surface morphological surveys for the undoped and the 0.1% doped lithium aluminium borate samples were carried out using the SEM image shown in Figure 2. The grains spread irregularly over the entire surface for both samples. The undoped sample was composed of some spongy clusters, while the 0.1% magnesium doped lithium aluminium borate showed aggregates with a bigger surface area which was composed of tiny particles that were smaller than that of the undoped samples.

**Figure 3:**
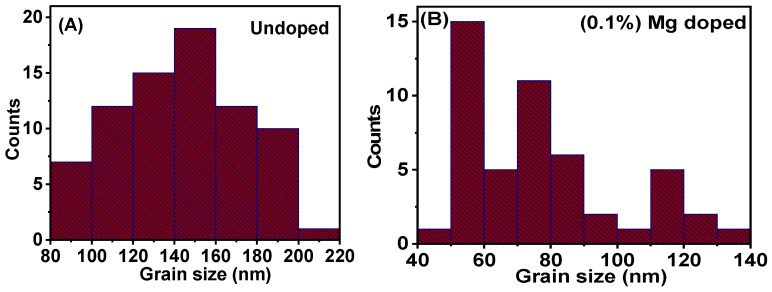
Grain size distribution plots for (**A**) undoped lithium aluminium borate and (**B**) 0.1% magnesium-doped lithium aluminium borate.

Size distributions from the cross-sectional surface morphology of the samples were further studied by usingimagej software which detected grain sizes that were presented as histogram plots, as shown in Figure 3. The undoped samples of lithium aluminium borate grain sizes were obtained from 80 to 220 nm with a maximum count of 19. The bar with the highest count was observed at grain sizesbetween 140 and 160 nm, while other bars appeared to decrease on either side of it. Magnesium (0.1%) doped lithium aluminium borate grain sizes were also obtained in a range from 40 to 140 nm with the highest count of 15. The highest bar, which indicates the highest number in the distribution, was observed to be at a grain size in the range of 50 to 60 nm. The grain size display of the magnesium-doped lithium aluminium borate was smaller than the result obtained from the distribution. Materials with size distributions in the range from 75 nm to 200 nm [33] are reported to have unique advantage when they are used as radiative and optical instruments.

### 3.3. Optical Properties

The energy required to stimulate an electron through a gap between the valence and the conduction band (also known as the energy band gap) and the interaction of the incident photon determines the optical properties of a phosphor material. These are important features of any semiconductor or phosphor material that is studied to determine structural, luminescence, thermal, and electronic properties.

**Figure 4:**
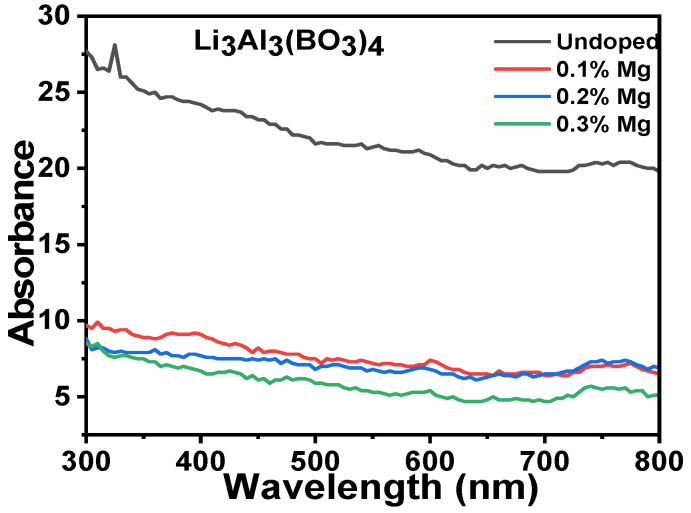
The optical absorbance plots versus the wavelength for the undoped concentrations and various concentrations of magnesium-doped lithium aluminium borate.

Figure 4 shows the absorbance plots for both the undoped concentrations and the various concentrations of magnesium-doped lithium aluminium borate. The absorbance of the materials was considered within the range of visible spectrum from 300 to 800 nm. The absorption spectrums observed were lines of slight peaks with absorbance that is high at the short wavelength and low at the long wavelength. The undoped lithium aluminium borate gave the highest absorbancewhen compared with the doped samples, as indicated in the plot in Figure 4. The doped samples of magnesium lithium aluminium borate decreased from0.1% to 0.3% mole percent concentration and increased from 0.4% to 0.5% (not shown). This shows that different minute values of magnesium-doped samples of lithium aluminium borate resulted in a slight decrease in the absorbance, which obtained saturation at 0.3% mole concentration.

**Figure 5:**
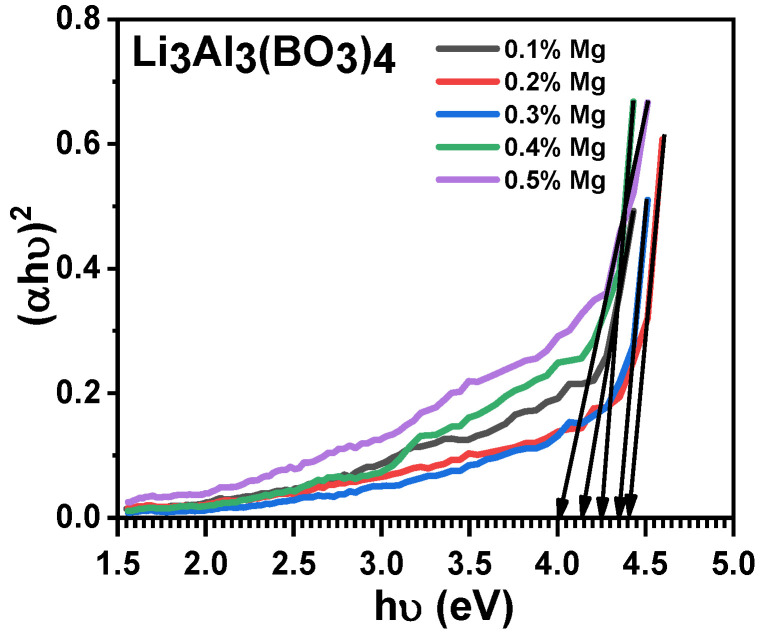
The energy band plot for the different concentrations of magnesium-doped lithium aluminium borate.

The band gap energy plot for each synthesized magnesium-doped lithium aluminium borate phosphor material is shown in Figure 5. This was achieved by using the stated equation [34]:

(2) $$\alpha \;hv=A{\left(hv-E_{g}\right)}^{n}$$

where the photon energy, transition probability, absorption coefficient, and Planck’ s constant are represented in the equation as *hv*, *A*, *α*, and *h*, respectively. The exponent n is the transition during the absorption process which can be values 1/2, 3/2, 2, and 3 which categorically represent the direct allowed, direct forbidden, indirect allowed, and indirect forbidden transition state. The linear extrapolation of the plots from the tail end to where it meets a point on the horizontal axis is denoted as the band gap energy. The direct allowed transition state from the energy band gaps obtained were observed at points on the horizontal axis in the plot in Figure 5 as 4.00, 4.15, 4.25, 4.35, and 4.40 eV, which corresponded to dissimilar mole percentages of Mg-doped lithium aluminium borate at 0.5%, 0.4%, 0.3%, 0.2%, and 0.1%, respectively. There was no regular order in the energy band gap regarding the increase in the mole percent of Mg-dopant used on the lithium aluminium borate. The undoped lithium aluminium borate phosphor reported so far had a band gap energy of 3.0 eV [5,35]. The use of Mg as a dopant increased the band gap energy of lithium aluminium borate phosphor, which makes it a good semiconductor that will display a luminescence phenomenon when it is used for dosimetry [5]. The majority of materials used for dosimetric applications have a high energy band gap. Based on reports, TL phosphorsthat are useful in radiation dosimetry, such as Al_2_O_3_ and Li_2_B_4_O_7_, have band gap energies of 9.5 eV and 7.5 eV, respectively [36]. The highest energy band gap ever obtained from doped lithium aluminium borate phosphor was 4.4 eV [5], which is also similar to our result.

### 3.4. Thermal Gravimetric Analysis (TGA)

**Figure 6:**
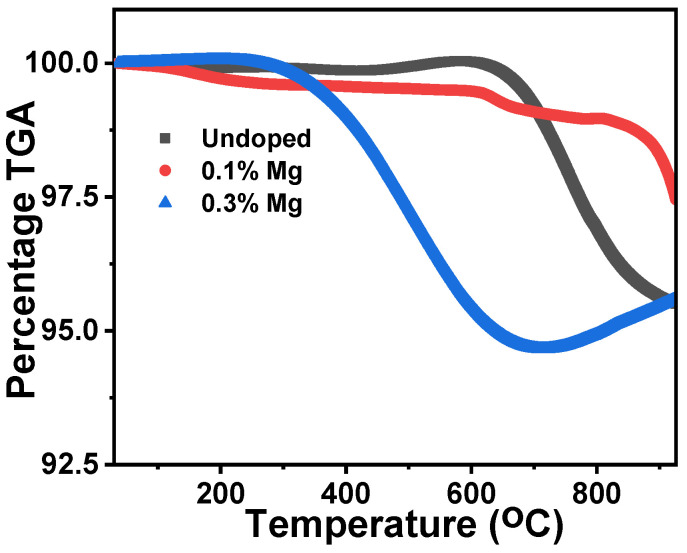
Thermal gravimetric analysis plots of undoped samples and some percentage compositions of Mg-doped lithium aluminium borate samples.

Thermal gravimetric analyses of the powdered samples of the undoped, 0.1% Mg, and 0.3% Mg-doped lithium aluminium borate were studied within a temperature range of 0 to 930 °C, as shown in Figure 6. A sharp decrease in the TGA (%) peak which exhibited a weight loss for the undoped, 0.1% Mg, and 0.3% Mg-doped lithium aluminium borate materials was observed at temperatures of 611, 830, and 308 °C, respectively. However, the 0.1% Mg-doped sample had a gradual decrease which started from the origin to the temperature of 830 °C where the sharp drop was seen. The samples will show some form of stability when used in any form of application at such a range observed before a drastic weight loss. María et al. [37,38] reported that weight loss or water loss during the thermal treatment of samples was a result of the decomposition of the boric acid (H_3_BO_3_ to B_2_O_3_) used during its preparation.

### 3.5. Thermoluminescence

Semiconductor materials undergo a stimulated process known as thermoluminescence (TL) after the absorption of energy from ionizing radiation [35]. TL was used to study how the phosphor materials were able to store the absorbed energy for a given period before discharging it in the form of visible light. The level of defect caused by a phosphor material also determines the TL intensity, which is usually displayed as a glow curve.

**Figure 7:**
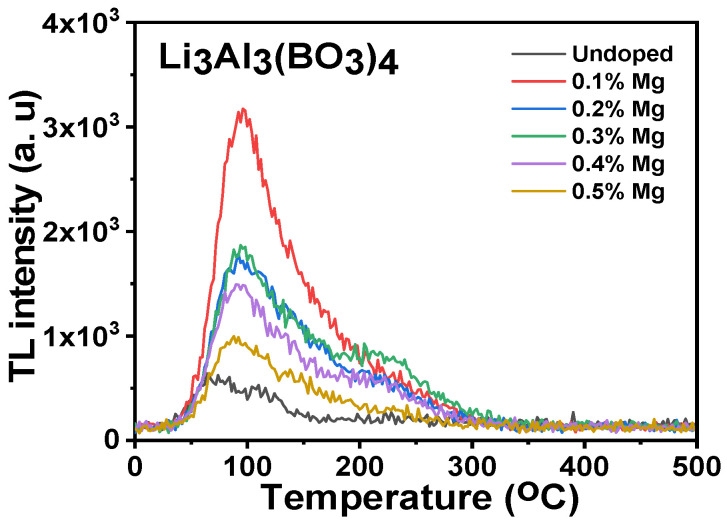
Undoped and Mg-doped lithium aluminum borate TL glow curves.

The glow curves obtained for the undoped samplesand the magnesium-doped lithium aluminium borate were heated from 0 to 500 °C, as shown in Figure 7. The plots were considered to be a very low dose rate (5 Gy) with major peaks at 86 °C as well as minor peaks observed at a higher temperature point from the glow curves. The glow curves were measured for the undoped concentrations and the various concentrations of 0.1, 0.2, 0.3, 0.4, and 0.5 percent mole of Mg-doped lithium aluminium borate. The undoped and 0.1% mole Mg-doped lithium aluminium borate were found to have the lowest and highest intensity peaks, while the intensity peaks of other mole concentrations were found to be intermediate. There was a corresponding order in the glow peaks which showed that as the concentration of Mg dopant enhanced, a decline in the peak intensity resulted, except for the 0.2% and 0.3% mole concentrations of Mg that appeared to have almost the same glow curve at their peaks. The glow curve trend obtained for the various concentrations of Mg-doped lithium aluminium borate compared well with that of its crystallize size and its optical energy band gap. This shows that an increase in the crystallite size of the samples resultedin a decrease in the glow curve for the various Mg-doped lithium aluminium borates. The decrease in the mole concentration of the magnesium used as a dopant on lithium aluminium borate resulted in a decrease in its optical energy band gap. The 0.2% and 0.3% Mg-doped lithium aluminium borate glow curves were similar because there were few differences compared to the crystallite size results and the optical energy band gap results reported earlier. Further research was carried out regarding the dosimetric properties by using only the 0.1% mole concentration of Mg-doped lithium aluminium borate because its high TL intensity resulted in a higher sensitivity of the material used for different dosimetric applications.

**Figure 8:**
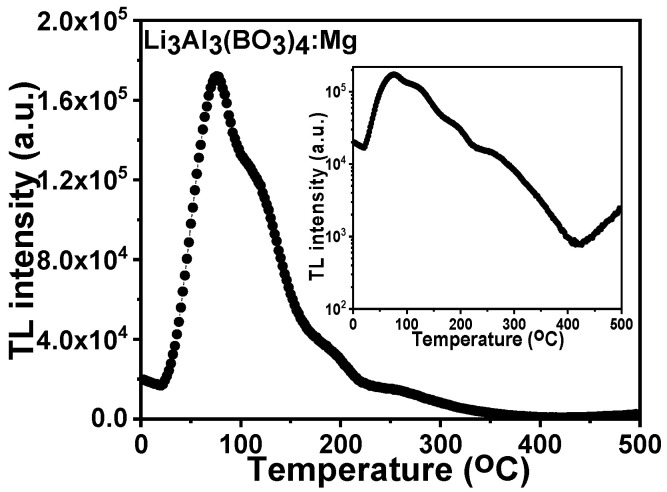
The TL glow curve of 0.1% mole Mg-doped lithium aluminium borate that was irradiated at 50 Gy. The inset identifies the supplementary peaks other than the foremost glow peak.

The TL glow curve for the 0.1% mole Mg-doped lithium aluminium borate was irradiated at 50 Gy after it was thermally cleaned and is shown in Figure 8. Thermal cleaning was done to eliminate the minor glow peaks that were present alongside the high temperature region. The inset in Figure 8 represents the logarithmic glow curve plot which was used to identify supplementary peaks other than the foremost glow peak.

**Figure 9:**
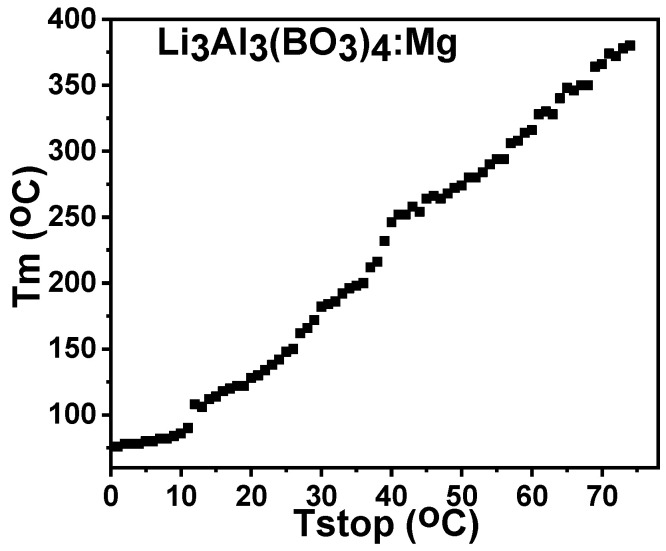
The Tm versus Tstop plot for the 0.1%Mg-doped lithium aluminium borate.

Figure 9 displays a plot of T_max_ alongside T_stop_ estimates with corresponding rangesin 1 °C intervals for Mg-doped lithium aluminum borate. The plot does not show a linear response; however, there was an increase in Tm with Tstop, which was a consequence of partial heating at various points along the glow curve. This demonstrates a second order thermoluminescence which results from overlapping peaks in the TL glow curve.

### 3.6. Kinetic Analysis

The activation energy, frequency factors, and order of kinetics of lithium aluminum borate doped with 0.1 percent magnesium were evaluated further by adopting the initial rise, variable heating rate, and deconvolution approach to extract data associated with the charge transfer process from the main glow peak. The glow curve was not isolated; therefore, these approaches were confirmed to be the only ones that were appropriate for the analysis.

#### 3.6.1. Initial Rise Method

**Figure 10:**
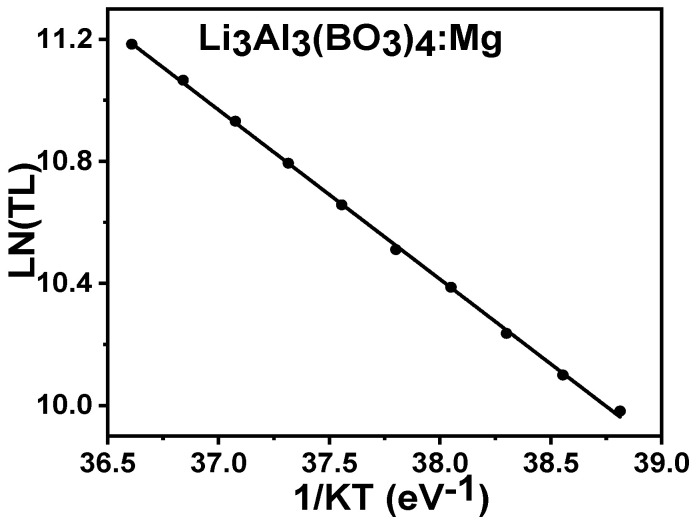
The plots of LN(TL) versus 1/kT for Mg-doped lithium aluminium borates.

The 0.1% magnesium-doped lithium aluminum borate materials were irradiated at a low dose of 50 Gyand were studied using the initial rise approach which was only applied at the lower peak temperature region. The plot of the natural log of *TL* intensity versus 1/*kT* revealed the activation energy E of the sample of lithium aluminium borate doped with magnesium. The experimental plot revealed the activation energy from the given equation.

(3) $$\log\;TL-\log C=\left(\frac{E}{kT}\right)$$

where *k*, *E*, *T*, and *C* represent the Boltzmann constant, activation energy, temperature, and constant [39]. The plots of the log of *TL* versus 1/*kT* for the Mg-doped lithium aluminum borate are shown in Figure 10, and the results from the analysis determined that the activation energy was 0.65 ± 0.01 eV.

#### 3.6.2. Curve Deconvolution

**Figure 11:**
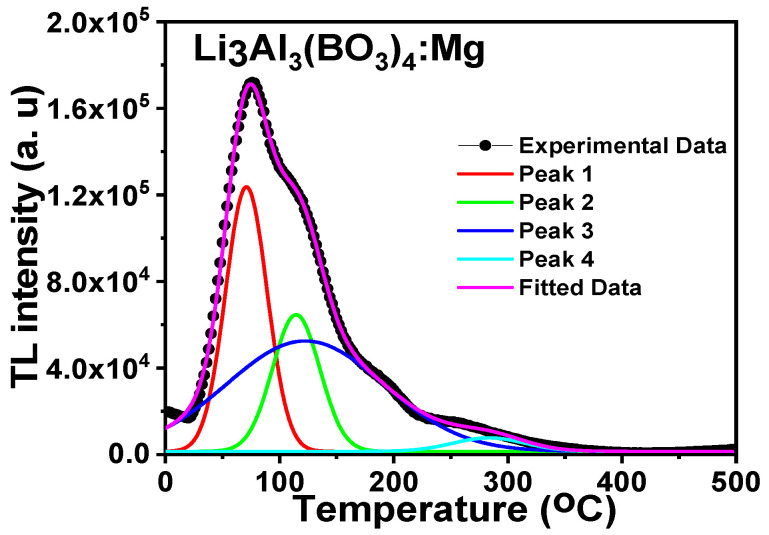
The glow curve deconvolution plot of Mg-doped lithium aluminium borate.

As shown in Figure 11, the curve deconvolution method was applied to the peak of the magnesium-doped lithium aluminium borate sample. Evaluation of the glow curve was achieved using the general order equation given by Kitis [40].

(4) $$I(T)=I_{m}b^{\frac{b-1}{b}}\exp\left(\frac{E}{kT}\frac{T-T_{m}}{T_{m}}\right){\left[(b-1)(1-\frac{2kT}{E})\frac{T^{2}}{T_{m}^{2}}\times \exp\left(\frac{E}{kT}\frac{T-T_{m}}{T_{m}}\right)+1+\left(b-1\right)\frac{2kT_{m}}{E}\right]}^{\frac{-b}{b-1}}$$

where *Im*, *Tm*, and *b* stand for the peak maximum intensity, the temperature at its peak maximum position, and the kinetic order. All other parameters in this equation have already been established. The figure of merit (FOM) was used to evaluate the quality of the glow curve fit, which was specified in the preceding equation as:

(5) $$\mathrm{FOM}=\frac{\sum_{T}\left|D_{\exp}-E_{\mathrm{fit}}\right|}{\sum_{T}E_{\mathrm{fit}}}\times 100\%$$

where E_fit_ and D_exp_ are the fitted data and experimental data, respectively. If the FOM is less than 3.5 percent, it is assumed that the fit is satisfactory [41]. The activation energy, frequency factor, and order of kinetic for the samples of Mg-doped lithium aluminum borate were estimated to be 0.69 + 0.01 eV, 1.72 × 10^9^·s^−1^, and 1.32, respectively. The obtained value for the activation energy was close to the one from the initial rise approach. The fit was considered adequate because the figure of merit for the Mg-doped lithium aluminum borate was 1.49%.

#### 3.6.3. Variable Heating Rate (VHR) Method

**Figure 12:**
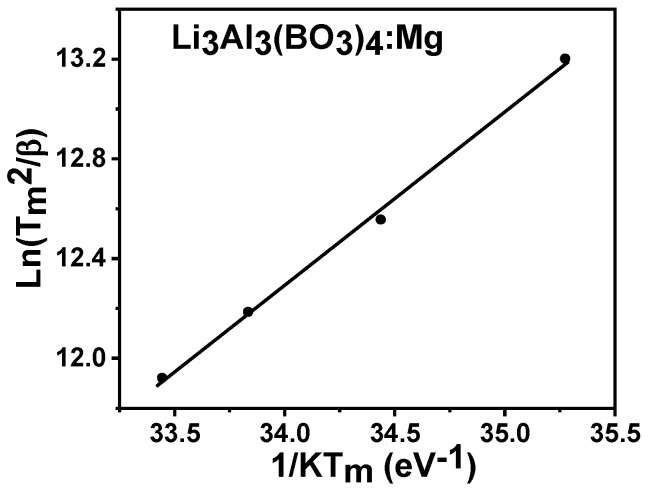
The heat rate plot for Mg-doped lithium aluminium borate.

The activation energy was determined by changing the glow curve heating rate. The analysis was based on the temperature alteration at intensity peaks (Tm) with various heat rates (0.2, 0.4, 0.6, and 0.8 °Cs^−1^) from a specified range that was less than one. The following equation was used to assess the data.

(6) $$In(T_{m}^{2}/\beta )=In(E/sk)+E/kT_{m}$$

where the frequency factor is represented as s and all other parameters have already been discussed [37]. The activation energy was 0.70 + 0.03 eV, and this was based on the straight line plot of *In*($T_{m}^{2}$/*β*) versus (1/*kTm*) in Figure 12. The frequency factor s was estimated based on the intercept as given in *In*(*E*/*sk*) when the extrapolation of 1/*Tm* was zero. The frequency factor s for the Mg-doped lithium aluminum borate yielded a value of 4.3 × 10^8^ s^−1^.

### 3.7. Dose–Response

**Figure 13:**
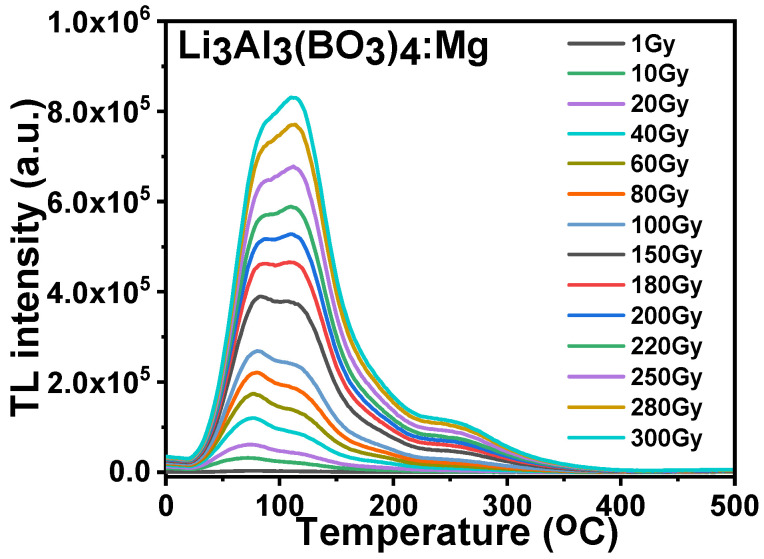
The dose–response of Mg-doped lithium aluminium borate.

Figure 13 illustrates the radiation dose–response of the magnesium-doped lithium aluminum borate materials at doses that ranged from 1 to 300 Gy. An increase in the TL main glow peak was detected at different levels of radiation exposure to the materials. A shift in the glow peak from 86 °C at a low radiation dose to 110 °C at a high radiation dose was observed as shown in the plot, and both peak temperatures had an almost equal intensity at 180 Gy. The reason for this is unknown; however, it could be due to structural deformation caused by the addition of the magnesium dopant into the compound of lithium aluminium borate.

**Figure 14:**
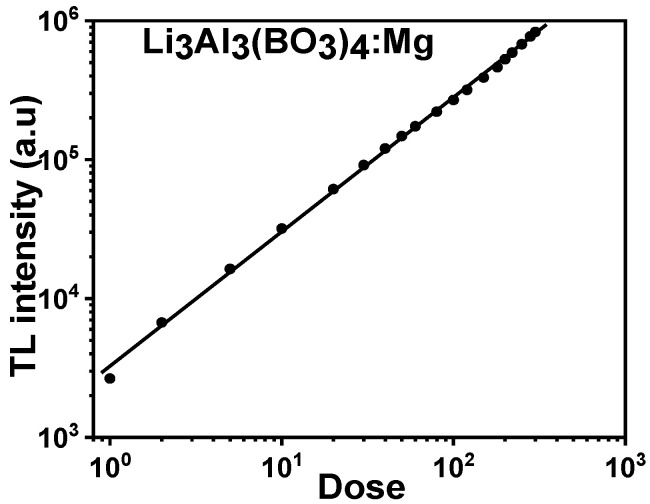
Dose linear–response for Mg-doped lithium aluminium borate.

The stated mathematical equation below was used to express the dose–response:

(7) LnTL = Lna + bLnD

where *TL* is the highest intensity, *D* is the recorded dose, and a and b are measured constants [39]. The slope obtained from the plot of the log of *TL* versus the log of *D* is equal to *b*, and the straight line depicts the linear dose–response for the Mg-doped lithium aluminium borate sample, as shown in Figure 14. The linear dose–response of Mg-doped lithium aluminium borate phosphor was observed in the given plot. As the observation shows, calibrations are not needed because the linear *TL* response for phosphor materials is useful and convenient fordosimetric materials for personnel and environmental monitoring.

## 4. Conclusions

The influence of magnesium doping on the structural, optical, and thermoluminescence of lithium aluminium borate was studied. A solid-state sintering technique was employed in the synthesis of borate samples. The crystallite and particle sizes obtained from the doped and undoped samples varied when they were compared. The optical absorbance and the energy band gap obtained from the direct transition varied for both the Mn-doped and the undoped lithium aluminium borate phosphor materials. The decrease in the TGA (%) peak exhibited a weight loss for the undoped, 0.1% Mn, and 0.3% Mn-doped lithium aluminium borate materials. The main glow peak was observed at 86 °C at a heat rate of 1 °C·s^−1^ after irradiation to 50 Gyalongside three other peaks which occurred at an exalted temperature level of the glow curve. The activation energy from the main glow peak was studied using initial rise, deconvolution, and variable heating rate approaches. The results obtained were consistent based on the methods that were adopted. A shift in the glow peak from 86 to 110 °C was observed as Mg-doped lithium aluminium borate material was exposed to radiation doses from 1 to 300 Gy. This implies that the structural performance of the lithium aluminium borate material improved due to the use of magnesium as a dopant and thatthe increase in TL sensitivity regarding the range of dose also improved.

## Figures and Tables

**Table 1:** The crystallite sizes for the samples of doped and undoped lithium aluminium borate.

Samples of Li_3_Al_3_(BO_3_)_4_	FWHM (°)	S (nm)
Undoped	0.15096	53.95
Mg (0.1%)	0.37878	34.85
Mg (0.2%)	0.19061	42.82
Mg (0.3%)	0.18364	45.24
Mg (0.4%)	0.11169	62.87
Mg (0.5%)	0.12084	67.35
